# Ultrasound-detected pathologies cluster into groups with different clinical outcomes: data from 3000 community referrals for shoulder pain

**DOI:** 10.1186/s13075-017-1235-y

**Published:** 2017-02-10

**Authors:** Gui Tran, Elizabeth M. A. Hensor, Aaron Ray, Sarah R. Kingsbury, Philip O’Connor, Philip G. Conaghan

**Affiliations:** 1 Institute of Rheumatic and Musculoskeletal Medicine, University of Leeds, Chapel Allerton Hospital, 2nd Floor, Chapeltown Road, Leeds, LS7 4SA UK; 2 NIHR Leeds Musculoskeletal Biomedical Research Unit, University of Leeds, Leeds, UK; 3 Arthritis Research UK Centre for Sport, Exercise and Osteoarthritis, Nottingham, UK

**Keywords:** Shoulder pain, Ultrasound scan, Pain assessment and management, Soft tissue rheumatism, Biomarkers

## Abstract

**Background:**

Ultrasound is increasingly used to evaluate shoulder pain, but the benefits of this are unclear. In this study, we examined whether ultrasound-defined pathologies have implications for clinical outcomes.

**Methods:**

We extracted reported pathologies from 3000 ultrasound scans of people with shoulder pain referred from primary care. In latent class analysis (LCA), we identified whether individual pathologies clustered in groups. Optimal group number was determined by the minimum Bayesian information criterion. A questionnaire was sent to all patients scanned over a 12-month period (*n* = 2322). Data collected included demographics, treatments received, current pain and function. The relationship between pathology-defined groups and clinical outcomes was examined.

**Results:**

LCA revealed four groups: (1) bursitis with limited inflammation elsewhere (*n* = 1280), (2) bursitis with extensive inflammation (*n* = 595), (3) rotator cuff tears (*n* = 558) and (4) limited pathology (*n* = 567). A total of 777 subjects (33%) completed questionnaires. The median (IQR) duration post-ultrasound scan was 25 (22–29) months. Subsequent injections were most common in groups 1 and 2 (groups 1–4 76%, 67%, 48% and 61%, respectively); surgery was most common in group 3 (groups 1–4 23%, 21%, 28% and 16%, respectively). Shoulder Pain and Disability Index scores were highest in group 3 (median 48 and 30, respectively) and lowest in group 4 (median 32 and 9, respectively). Patients in group 4 who had surgery reported poor outcomes.

**Conclusions:**

In a community-based population, we identified clusters of pathologies on the basis of ultrasound. Our retrospective data suggest that these groups have different treatment pathways and outcomes. This requires replication in a prospective study to determine the value of a pathology-based classification in people with shoulder pain.

**Electronic supplementary material:**

The online version of this article (doi:10.1186/s13075-017-1235-y) contains supplementary material, which is available to authorized users.

## Background

Shoulder pain is common, with an estimated worldwide incidence of 0.9% to 2.5% and point prevalence of up to 26% [1]. Importantly for our ageing society, shoulder pain increases with age, with a prevalence of 21% in those over 70 years old [2]. In the United Kingdom, approximately 4% of adults consult their general practitioner (GP) about shoulder pain [3, 4]. Management remains challenging and often results in poor outcomes. Despite the short-term beneficial effects of community-based interventions, 50% of people continue to have pain at 18 months, though pain can also follow a remitting-relapsing course over time, making evaluation of treatment at single time points difficult [5, 6]. Shoulder pain has a significant impact on quality of life, and the economic burden has been estimated to be €689 per person annually [7, 8].

The clinical diagnosis of shoulder pathologies is difficult, and evidence suggests poor levels of reliability and reproducibility amongst clinicians when examining shoulder pain [9, 10]. Recent qualitative research has shown uncertainty amongst GPs in the diagnosis of patients with shoulder pain [11]. Diagnosis is complicated further by inconsistent nomenclature and classifications [12]; pathologies of the rotator cuff (RC) have been described by a variety of terms, including *RC syndrome*, *shoulder impingement syndrome*, *subacromial bursitis* and *RC tendinitis*.

Ultrasound offers accurate detection of pathology, and, in the context of the reported diagnostic uncertainty, it is unsurprising that its use is increasing. From 2001 to 2009 in Australia, there was a fourfold rise in shoulder ultrasound scans [13]. The number of primary care referrals for shoulder ultrasound scans in a U.K. regional centre tripled to 3000 between 2007 and 2015 (Leeds Teaching Hospitals NHS Trust, unpublished data). However, it is still unclear how information from ultrasound relates to treatment and long-term outcomes [14, 15]. The extent to which diagnostic tests on shoulder pain inform and affect patient management and outcomes has been highlighted previously in several systematic literature reviews as an area that needs to be investigated [13, 16].

Shoulder pain may have complex aetiologies, and pathologies often do not occur in isolation; some may respond to particular therapies better than others, which could complicate assessment of efficacy if they co-occur. Therefore, understanding patterns of shoulder pathology may help in targeting therapies more effectively. Given the uncertainty in clinical diagnosis, it seems reasonable to examine the potential of pathology-based diagnosis using ultrasound. If ultrasound cannot identify groups of patients who will achieve different outcomes, either in the current care pathway or in trials of targeted therapies, there would be limited justification for its continued use in this patient group. As a first step toward understanding the importance of a pathology-based classification, we aimed to determine whether distinct clusters of ultrasound-defined pathologies exist and whether there is any evidence that these have implications for long-term clinical outcomes.

## Methods

### Patients

Ultrasound reports were retrieved for consecutive primary care patients referred to a single centre’s radiology department (Chapel Allerton Hospital, Leeds, UK) for a scan of their shoulder. We included 3000 patients on the basis of estimated annual referrals and included scans between 2012 and 2013. Inclusion criteria were that patients be aged over 18 years, referred from primary care, referred for shoulder pain and attending for their first ultrasound scan. Patients were excluded if they had had previous surgery or had not been referred from primary care. These patients were identified by details on the referral card and electronic patient records.

In our centre, guidelines advise that patients be referred from primary care for an ultrasound scan if they have moderate-severe painful abduction, have not improved after physiotherapy or have suspected acromioclavicular joint (ACJ) pain. However, clinical experience of the broad range of presenting symptoms and signs raises questions about adherence to these recommendations and may further highlight discrepancies in clinical evaluation of shoulder pain.

### Ultrasound scans

Data from a single shoulder per patient were utilised, and, where identifiable, the first symptomatic shoulder was included. Eligible patient records were examined to identify the first ultrasound scan for a selected shoulder (even if the first scan fell outside the collection dates). Scans were obtained by musculoskeletal radiologists and sonographers. Previous work has shown that inter-rater reliability for shoulder pathologies between two of the radiologists is substantial (all κ values >0.6) for full-thickness RC tear, calcification, impingement and tendon abnormalities [17]. The following features were documented as present or absent: bursitis, impingement, calcific tendinitis, ACJ degeneration, glenohumeral osteoarthritis, adhesive capsulitis, biceps tenosynovitis, RC tendinopathy and full or partial RC tear (see Additional file 1 for definitions). After discussion with the sonographers, impingement was assumed to be present if there was a full-thickness RC tear, even if impingement was not reported. Other pathologies were assumed to be absent if not reported. Other details recorded included age at time of scan, sex, whether an injection was given on the day of the scan, and (where available) duration of pain.

### Questionnaire

A postal questionnaire was sent to all eligible patients scanned in 2013. A second wave of questionnaires was sent to those who had not replied after 4 weeks. Data collected included demographics; characteristics of pain; previous treatment; Shoulder Pain and Disability Index (SPADI), a validated self-administered questionnaire used to measure pain and disability in the shoulder in the past week [18–20]; EuroQol five dimensions scale (EQ-5D-5 L), a self-report measure used to define health status [21]; Marx shoulder activity scale [22]; and self-reported comorbidities. Returned questionnaires were matched to ultrasound findings.

### Statistical analysis

Latent class analysis (LCA) was performed (see Additional file 1 for full details). LCA identifies clusters which group together people who share similar characteristics, in this case people who share a distinct pattern of shoulder pathology. The model calculates the probability of group membership for each person and assigns each individual to the group with the highest probability; the accuracy of the classification can be improved by including covariates such as age and sex. The classes we identified (hereafter *groups*) were then compared for age, sex, duration of pain (according to the initial scan record), injection (according to the initial scan record) and the presence of each pathology. To check for responder bias, these details were also compared between patients who completed the questionnaire and those who did not. Questionnaire responses were compared between the pathology groups using quantile, Poisson or binary logistic regression, according to the outcome type, adjusting for age and sex. Appropriate checks were made that the data satisfied the test assumptions. We used Stata version 13.1 software (StataCorp, College Station, TX, USA) for analysis.

## Results

To identify 3000 eligible ultrasound scans, 3035 referral cards were reviewed. Reasons for exclusion included 6 scans technically difficult, 9 scan results inaccessible, 17 with soft tissue lumps examined and 3 with guided procedures without diagnostic scans.

### Ultrasound pathologic findings

In the 3000 patients selected, the mean age was 54.6 years, and 52% were female (Table 1). For eight patients, impingement could not be assessed owing to difficulty in moving the patient’s arm; impingement status was set to ‘missing’ for these patients. The most common pathologies were subacromial impingement (69%) and bursitis (68%), followed by ACJ degeneration (40%), tendinopathy (36%), calcific tendonitis (12%), biceps tenosynovitis (7%), glenohumeral osteoarthritis (6%) and adhesive capsulitis (3%).

**Table 1:** Demographics and ultrasound findings in patients included in the full scan review and those sent a questionnaire

	Full scan review (*n* = 3000)	Questionnaire recipients (*n* = 2322)	Responders (*n* = 777)	Non-responders (*n* = 1545)
Age, years, mean (SD)	54.6 (15.1)	54.1 (15.1)	56.4 (13.8)	53.0 (15.6)
Female sex, %	52%	52%	54%	51%
Pain duration, months, median (IQR)	5 (3–9), *n* = 1165	5 (3–10), *n* = 868	5 (3–10), *n* = 292	5 (3–9), *n* = 576
Steroid injection at time of scan, %	33%	31%	37%	28%
RC tear, %	26%	24%	27%	23%
Full thickness RC tear, %	19%	18%	20%	17%
Bursitis, %	68%	71%	72%	71%
Impingement, %	69% (of 2992)	68% (of 2314)	71% (of 776)	67% (of 1538)
Calcific tendinitis, %	12%	12%	14%	12%
ACJ degeneration, %	40%	45%	48%	43%
Glenohumeral OA, %	6%	5%	5%	6%
Adhesive capsulitis, %	3%	3%	4%	3%
Biceps tenosynovitis, %	7%	9%	9%	9%
Rotator cuff tendinopathy, %	36%	38%	40%	37%

*ACJ* Acromioclavicular joint, *OA* Osteoarthritis, *RC* Rotator cuff

### Latent class analysis

The LCA suggested four or five groups existed. (For full details, see Additional file 1: Tables S1–S3.) We retained the four-group solution. On the basis of patterns of pathology in each group, we interpreted that they represented bursitis with limited inflammation elsewhere (group 1), bursitis with extensive inflammation (group 2), RC tears (group 3) and limited pathology (group 4) (Table 2).

**Table 2:** Demographic characteristics and ultrasound pathology findings in each of four pathology groups

	Bursitis (limited inflammation) (*n* = 1280)	Bursitis (extensive inflammation) (*n* = 595)	RC tear (*n* = 558)	Limited pathology (*n* = 567)
Percentage of sample	43%	20%	18%	19%
Age, years, mean (SD)	47.6 (11.5)	64.2 (10.5)	69.1 (11.2)	46.1 (13.5)
Female sex, %	54	52	51	46
Pain duration, months,^a^ median (IQR)	5 (3–10)	5 (3–9)	5 (3–9)	4 (3–8)
Steroid injection at time of scan, %	44	37	13	26
RC tear, yes/no, %	2	24	100	6
Full-thickness RC tear, %	1	13	86	<1
Bursitis, %	100	94	30	7
Impingement, %	88	65	91	6
Calcific tendinitis, %	14	18	5	9
ACJ degeneration, %	26	83	54	15
Glenohumeral OA, %	<1	10	16	2
Adhesive capsulitis, %	<1	3	<1	12
Biceps tenosynovitis, %	<1	25	10	2
Rotator cuff tendinopathy, %	23	92	24	20
Probability of membership, mean	0.88	0.80	0.89	0.93

*ACJ* Acromioclavicular joint, *OA* Osteoarthritis, *RC* Rotator cuff

^a^ *n* = 507, 198, 233 and 227 in groups 1–4, respectively

**Fig. 1:**
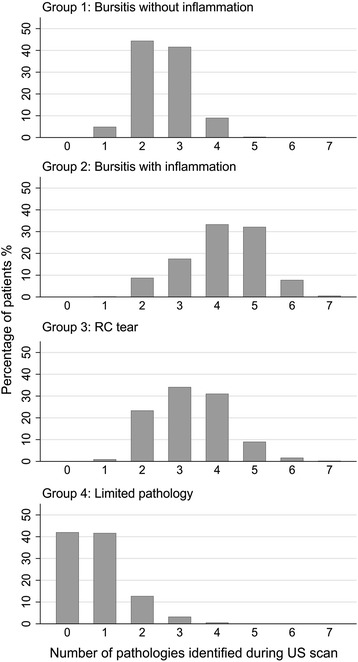
Pathology count by group. *RC* Rotator cuff, *US* Ultrasound

Group 1 was the largest (43%) (Table 2); the other three groups each represented approximately 20%. The groups were similar in gender balance or duration of pain prior to the first scan. Patients in group 4 were the youngest; 42% had no pathologies recorded, and a further 42% had just one pathology reported (Fig. 1). In group 4, mean age was similar to group 1; all patients in group 1 had bursitis, but few had tendinopathy or ACJ degeneration. In group 2, on average 20 years older than groups 1 and 4, almost all patients had bursitis, RC tendinopathy and ACJ degeneration, and one-fourth had biceps tenosynovitis. Patients in group 3 were the oldest on average; all had RC tears, which were full-thickness tears in the majority. Patients in this group had the highest rate of glenohumeral osteoarthritis, as might be expected for their age, but a smaller proportion in group 3 than in group 2 had ACJ degeneration. Nearly all patients in group 3 had impingement; however, comparatively few had bursitis compared with groups 1 and 2.

### Patient questionnaire findings

A postal questionnaire was sent to all eligible patients scanned in 2013 (*n* = 2322). Of these patients, 777 completed questionnaires (33%), and we received replies from a further 233 (10%) who declined to participate. Responders and non-responders were similar in gender balance, age and ultrasound pathology findings (Table 1). Some respondents reported a diagnosis of rheumatoid arthritis (RA) (*n* = 87). Ultrasound findings did not show intra-articular synovitis in the RA-reporting group. Re-running the LCA in questionnaire respondents who did not report having RA (*n* = 690) resulted in very results similar to those of the full (*n* = 3000) LCA (see Additional file 1: Table S4 for details). We therefore retained the original pathology groupings; however, in the analyses of questionnaire outcomes, we excluded those reporting RA.

Questionnaires were completed by 30%, 36%, 34% and 25% of patients in groups 1–4, respectively. Older patients were more likely to respond; there were no differences in adjusted response rates between groups (probability of response in group 1 = 0.31 [0.28–0.34], group 2 = 0.33 [0.29–0.37], group 3 = 0.30 [0.26–0.35], group 4 = 0.28 [0.23–0.32]; chi-square = 3.04, *p* = 0.386).

Questionnaire results for all respondents are presented in Additional file 1: Table S5; 67% still experienced pain at a median (IQR) of 25 (22–29) months since their scan. Ultrasound findings by group (restricted to questionnaire respondents) are provided in Additional file 1: Table S6. Follow-up duration was similar in the four groups (Table 3). Between 63% (groups 1 and 2) and 77% (group 3) of patients reported persistent pain at follow-up.

**Table 3:** Questionnaire outcomes summarised by pathology group, excluding those reporting rheumatoid arthritis (*n* = 690)

	Bursitis (limited inflammation) (*n* = 291)	Bursitis (extensive inflammation) (*n* = 177)	RC tear (*n* = 122)	Limited pathology (*n* = 100)
Percentage of sample	40%	39%	33%	42%
Age, years, mean (SD)	49.9 (11.1)	64.8 (9.2)	67.1 (10.4)	47.5 (12.6)
Female sex, %	63	45	47	55
Smoker, %	40	39	33	42
Comorbidity count, median (IQR)	1 (0–2)	2 (1–2)	2 (1–3)	1 (0–2)
Painful sites count (including target), median (IQR)	3 (1–6)	3 (1–7)	3 (1–6)	2 (1–4)
Follow-up, months, median (IQR)	26 (23–29)	26 (22–29)	25 (23–29)	24 (21–26)
Had shoulder fracture before scan, %	2	2	<1	1
Had shoulder dislocation before scan, %	1	–	4	1
Had breast/shoulder cancer before scan, %	<1	–	–	–
Had major injury to target shoulder before scan, %	7	8	18	3
Had shoulder fracture since scan, %	<1	1	–	1
Had shoulder dislocation since scan, %	<1	–	–	–
Had breast/shoulder cancer since scan, %	<1	<1	–	–
Had major injury to target shoulder since scan, %	1	2	7	1
Had physiotherapy since scan, %	65	62	59	58
Had injection since scan, %	76	67	48	61
Had more than one injection since scan, %	33	30	22	24
Had surgery since scan, %	23	21	28	16
Still has shoulder pain, %	63	63	77	64
If still in pain				
Pain duration, months, median (IQR)	24 (12–36)	21 (12–36)	25 (12–36)	24 (18–36)
Has pain-free periods, %	70	77	72	77
Experiences pain on moving in a certain way, %	91	93	92	85
If not still in pain				
How long since last had pain, months, median (IQR)	12 (6–17)	13 (12–20)	12 (6–18)	12 (10–20)
How long did pain last, months, median (IQR)	9 (3–15)	9 (5–18)	12 (12–18)	6 (4–12)
Symptoms at time of questionnaire				
SPADI pain, median (IQR)	34 (4–62)	26 (2–62)	48 (18–66)	32 (6–64)
SPADI difficulty, median (IQR)	13 (0–45)	14 (0–43)	30 (10–54)	9 (0–38)
SPADI total, median (IQR)	24 (3–52)	21 (3–51)	41 (15–59)	25 (5–49)
Shoulder activity score, median (IQR)	6 (3–10)	6 (3–10)	5 (3–9)	7 (4–11)
EQ-5D health index score, median (IQR)	0.8 (0.6–0.8)	0.7 (0.6–0.8)	0.7 (0.5–0.8)	0.8 (0.7–0.8)
EQ-5D VAS, median (IQR)	80 (55–90)	80 (60–90)	75 (60–80)	80 (70–90)
EQ-5D anxiety or depression (>0), %	34	30	40	29
Depression reported in comorbidity list, %	17	14	18	14
Difficulty standing from sitting (>1), %	16	20	21	10

*EQ-5D* EuroQol five dimensions scale, *SPADI* Shoulder Pain and Disability Index, *VAS* Visual analogue scale

Data were not available for all outcomes for all survey responders; see Additional file 1: Table S8 for numbers of patients with data available

The most commonly reported painful sites other than the target shoulder were lower back (36%), neck (33%), and knees either ipsilateral (27%) or contralateral (24%) to the target shoulder (Additional file 2: Figure S1). Adjusted estimates of the number of painful sites were highest in group 2 (mean 4.2) and lowest in group 4 (mean 3.0) (Table 4).

**Table 4:** Age- and sex-adjusted comparisons between pathology groups for key outcomes, excluding those reporting rheumatoid arthritis (*n* = 690)

	Bursitis (limited inflammation) (*n* = 291)	Bursitis (extensive inflammation) (*n* = 177)	RC tear (*n* = 122)	Limited pathology (*n* = 100)	Test result, *p* value
Number of painful sites^a^	4.0 (3.7–4.2)	4.2 (3.9–4.6)	3.6 (3.3–4.0)	3.0 (2.6–3.3)	χ^2^ = 29.0, *p* < 0.001
Steroid injection at time of scan	0.49 (0.42–0.55)	0.40 (0.32–0.47)	0.12 (0.06–0.18)	0.34 (0.24–0.44)	χ^2^ = 40.1, *p* < 0.001
Steroid injection since scan	0.76 (0.70–0.81)	0.67 (0.69–0.75)	0.49 (0.39–0.59)	0.63 (0.52–0.73)	χ^2^ = 20.3, *p* < 0.001
Physiotherapy	0.59 (0.53–0.66)	0.67 (0.60–0.75)	0.67 (0.58–0.76)	0.53 (0.43–0.63)	χ^2^ = 5.0, *p* = 0.171
Surgery	0.19 (0.15–0.24)	0.25 (0.18–0.33)	0.35 (0.25–0.46)	0.14 (0.07–0.21)	χ^2^ = 10.5, *p* = 0.015
Pain at follow-up	0.59 (0.53–0.65)	0.68 (0.61–0.75)	0.81 (0.74–0.88)	0.60 (0.50–0.70)	χ^2^ = 17.3, *p* < 0.001
SPADI pain^b^	29 (22–36)	32 (23–41)	51 (40–62)	28 (17–40)	*F*(3, 672) = 4.0, *p* = 0.008
SPADI difficulty^b^	15 (10–20)	19 (12–25)	31 (23–39)	11 (3–20)	*F*(3, 668) = 4.1, *p* = 0.006
SPADI total^b^	23 (18–29)	25 (18–32)	42 (34–51)	20 (11–29)	*F*(3, 666) = 5.3, *p* = 0.001

*RC* Rotator cuff, *SPADI* Shoulder Pain and Disability Index

All values in the table are adjusted probability of the outcome (95% CI) unless otherwise stated

^a^Adjusted mean (95% CI)

^b^Adjusted median (95% CI)

There was descriptive evidence that treatment differed according to presence of certain individual pathologies (Additional file 1: Table S7). There were clear differences in treatment at the group level for steroid injection (*p* < 0.001) and surgery (*p* = 0.015) (Table 4). Those in group 1 (bursitis with limited inflammation) were the most likely to have had steroid injection(s) (adjusted probability 76%), whereas those in group 3 (RC tears) were least likely (49%). Patients in group 3 were the most likely to have had surgery (35%); surgery was least likely in the limited pathology group (14%). In patients with bursitis, those in group 3 were less likely to have received a steroid injection at time of scan than those in groups 1 and 2 (estimated probability of injection [95% CI] group 1 = 0.48 [0.41, 0.54]; group 2 = 0.41 [0.33, 0.49]; group 3 = 0.20 [0.06, 0.33]; chi-square = 8.6, *p* = 0.014). Adjusted rates of physiotherapy did not differ between groups.

**Fig. 2:**
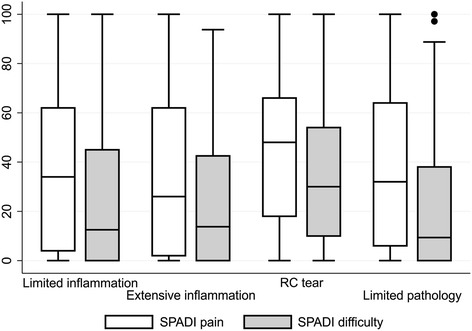
Shoulder Pain and Disability Index (SPADI) boxplot. *RC* Rotator cuff

Groups differed in the severity of their reported symptoms. Group 3 (RC tears) was more likely to still have pain at follow-up (Table 4), and subjects in this group reported the highest levels of SPADI pain. A similar trend was seen for SPADI difficulty scores (Tables 3 and 4; Fig. 2). These trends were not explained by the higher rate of surgery in group 3; those who had surgery reported lower scores (adjusted difference in median total score [95% CI] −19 [−38, 0]; z-score = −1.98, *p* = 0.048). The differences in total SPADI by surgery were negligible for group 1 (−8 [−22, 5]; z-score = −1.23, *p* = 0.220) and group 2 (0 [−17, 17]; z-score = −0.04, *p* = 0.965). In contrast, in group 4, total SPADI was substantively higher in the patients who had surgery (25 [−1, 51]; z-score = 1.89, *p* = 0.059) (see Additional file 3: Figure S2).

Patients in group 4 who had surgery were more likely to report a diagnosis of depression than those who had not (adjusted probability [95% CI] 0.40 [0.15–0.66] vs. 0.09 [0.02–0.15]), whereas surgery was not associated with depression in the other groups (interaction effect for group × surgery chi-square = 9.4, *p* = 0.025). However, the nature of the association between symptoms, surgery and depression cannot be determined in this cross-sectional study. There were no substantive age- and sex-adjusted differences between the groups in shoulder activity level, EQ-5D index score, EQ-5D health VAS score, the number of comorbidities reported, or the odds of reporting difficulty standing from sitting (data not shown).

## Discussion

This study has demonstrated, for the first time to our knowledge, clustering of ultrasound pathologies into four groups. These groups reported different treatments and to some extent had different age- and sex-adjusted outcomes at 2 years; however, owing to the low questionnaire completion rate, the longitudinal results need to be interpreted with caution.

Currently, there is limited evidence-based guidance on the role of imaging in the shoulder pain care pathway, and international guidance pre-dates the wide availability of ultrasound [23]. Though guidance for the diagnosis and management of many common painful musculoskeletal problems generally does not require imaging as part of routine care, the uncertainty in clinical evaluation, poor patient outcomes and increasing use of ultrasound support critical evaluation of the usefulness of a pathology-based classification. Researchers in a recent pragmatic randomised trial reported no evidence of difference in patient-perceived recovery between those with ultrasound-tailored treatment and usual-care groups [24]. Ultrasound-guided treatment was targeted at individual pathologies, and it would be interesting to see if outcomes would differ using our novel pathology-based classification.

The clinical validity of the pathology groups identified in this study require further evaluation in future studies. Conceivably, patients with just one pathology may respond differently to treatment compared with patients in whom the same pathology co-occurs with other pathologies. Although we did not attempt to examine the efficacy of different treatments, different patterns of treatment were reported. Group 1 was most likely to receive steroid injections. Steroid treatment may help with subacromial bursitis in the short term [25], which may explain the treatment in this group. Groups 1 and 2 may represent a spectrum; members of group 2 are older, and if we were to follow patients similar to those in group 1 over time, their patterns of shoulder pathology may eventually resemble those of group 2. Group 3 was the oldest group, confirming previous studies which have shown that RC tears increase with age [26]. Members of group 3 were less likely to receive steroid injections, even if they had concurrent bursitis, and they were more likely to undergo surgery. Steroid injections may impede tendon repair, and RC tears offer a surgical target, which may explain the variation in treatment. Group 3 also had the highest level of current pain and functional impairment. Surgical repair techniques of RC tears vary, and surgery has been shown to have conflicting results in improving outcomes in patients with shoulder pain [27–29]. Our data suggest that those who had surgery reported lower levels of pain and functional impairment. Group 4 was the youngest group, and a smaller proportion of these patients reported having surgery, because fewer had detectable pathologies present. Group 4 also had the lowest levels of pain and functional disability of all the groups.

Many (42%) in group 4 had no pathology; some of these patients may have improved at the time of their ultrasound scan. Another explanation is that other pathologies were present that ultrasound could not detect. Ultrasound is as sensitive and specific as magnetic resonance imaging (MRI) in detecting RC disorders [30], but further work is required to understand its sensitivity and specificity in detecting other pathologies, such as calcific tendinopathy. Furthermore, pathologies such as labral tears require MRI for identification [31, 32]. In addition, imaging-detected pathologies may not correlate with clinical findings. In the present study, 16% of patients without detectable pathology received steroid injections at the time of their scan; many reports documented that this was after discussion with the patient, and in some cases because clinical impingement was suspected even though this was not confirmed by the scan. A further explanation could be that the pain may be referred from other regions, such as the neck. The cause of chronic pain is multifactorial, and other features apart from imaging pathology play a role in characterising pain. Psychological factors such as fear avoidance, depression and poor quality of life can result in worse pain, function and perceived recovery outcomes [33, 34]. Ultrasound-detected pathologies have previously been reported in asymptomatic individuals, and further work is required to understand which factors result in the development and progression of symptoms in these individuals [14, 26, 27, 35].

Although we looked at associations between baseline pathologies and outcomes, the absence of baseline clinical data means we could not fully evaluate the predictive value of ultrasound. Previous attempts at identifying predictors of outcomes in people with shoulder pain have been made [36–40]. Pain characteristics such as worse baseline pain, duration of pain, concomitant psychological complaints, other concomitant musculoskeletal problems and repetitive shoulder action resulted in worse outcomes [37, 39–41]. Existing prognostic models to improve shoulder pain management have yet to be validated and assessed for clinical utility [39, 42]. There are very limited studies evaluating the prognostic role of ultrasound in shoulder pain: One suggested that the absence of subacromial bursa pathology may be a predictor of excellent outcomes at 3 weeks [36].

This study has a number of limitations. This study was undertaken in a single centre, though the sample size was large; the demographics of included patients seem similar to those of other large community cohorts [4, 43]. There was no control group, limiting our interpretation of pathologies and symptoms. Our local care pathway recommends that patients over the age of 65 years with shoulder pain undergo radiography of their shoulder, which may result in a channelling bias because patients with radiographic osteoarthritis may not undergo ultrasonography. In this study, local recommendations suggested that patients were referred for an ultrasound scan if they had moderate-severe pain and were not responding to physiotherapy, which could have led to selection bias in our cohort. However, it would seem that this group would likely be typical of patients with shoulder pain requiring investigation in potential future care pathways. Although the radiographers in this study followed a standardised method of performing ultrasonography of shoulders [44], standardised reporting of all pathologies was not routine, so if pathology was not documented, it was assumed absent. It is possible that some pathologies may not have been reported, especially if lesions that are considered more severe or clinically relevant are primarily reported. Group 3 had the highest level of glenohumeral osteoarthritis but a lower frequency of ACJ degeneration; the latter finding may be a result of non-standardised reporting, although it may also be an artefact introduced as a result of the groupings formed on the basis of LCA. A prospective study using standardised criteria for the different diagnostic labels is needed. This was a retrospective study, so we were unable to explore inter-reader reliability, especially in partial RC tears, where authors of a recent review showed that ultrasound has some difficulty in diagnosing this pathology [30]. Previous work has shown that, for most shoulder pathologies, the inter-rater reliability for two of the present sonographers was acceptable [17]. Impingement was assumed in all patients with complete RC tears. The patient questionnaire was retrospective, raising the possibility of recall bias. Only 33% completed the questionnaires; therefore, there is potential for selection bias. However, our work suggests that completers and non-completers were very similar in demographic characteristics and pathologic findings. Importantly, though we recorded symptoms around 2 years after an initial scan, we were unable to determine initial symptoms and subsequent changes. The prognostic value of a pathology-based classification needs to be established before consequent treatment pathways can be explored.

## Conclusions

This study demonstrates, for the first time to our knowledge, that patients undergoing ultrasound scans for shoulder pain can be grouped according to pathologic patterns. Our data suggest that these groups may receive different treatment and have different outcomes. These preliminary data support further exploration of the potential benefits of a pathology-based classification for shoulder pain.

## Additional files

Additional file 1: Supplementary materials, including ultrasound reporting definitions, LCA analysis and supplementary tables mentioned in the body of main text. (DOCX 33 kb)

Additional file 2: Figure S1. Joint pain reported. (JPG 2553 kb)

Additional file 3: Figure S2. Total SPADI by surgery. (JPG 2552 kb)

## Abbreviations

ACJ: Acromioclavicular joint
EQ-5D-5L: EuroQol five dimensions scale
GP: General practitioner
LCA: Latent class analysis
MRI: Magnetic resonance imaging
NIHR: National Institute for Health Research
OA: Osteoarthritis
RA: Rheumatoid arthritis
RC: Rotator cuff
SPADI: Shoulder Pain and Disability Index
US: Ultrasound
VAS: Visual analogue scale
