# Supplementary material for: Ultrasound-detected pathologies cluster into groups with different clinical outcomes: data from 3000 community referrals for shoulder pain
Source: Arthritis Res Ther. 2017 Feb 10;19:30. doi: 10.1186/s13075-017-1235-y (PMC5304553; doi:10.1186/s13075-017-1235-y)
Supplement: Supplementary file 1 — Supplementary materials, including ultrasound reporting definitions, LCA analysis and supplementary tables mentioned in the body of main text. (DOCX 33 kb) [file 13075_2017_1235_MOESM1_ESM.docx]

### Ultrasound reporting definitions for shoulders

#### RC Tears

Record foot print tears as partial thickness if articular surface not involved

#### Bursal thickening

Bursal effusion or synovitis >0.5mm

#### Dynamic subacromial impingement

Bunching of the bursa lateral to the coraco-acromial ligament during active abduction in the absence of rotator cuff tear.

#### Calcific tendinitis

Diagnosed in the presence of globular calcific deposition, exclude linear entheseal calcifications.

#### ACJ pathology

Diagnosed in the presence of osteophytosis or synovitis, effusion, joint malalignment and bone cortex irregularity.

#### Glenohumeral OA

Diagnosed by the presence of marginal osteophytosis at the cartilage bone junction or humeral articular cartilage thinning.

#### Adhesive Capsulitis/ frozen shoulder

Diagnosed in patients with limited passive external rotation possibly with a small LHB effusion and thickening of the coracohumeral ligament compared to the contra-lateral side in the absence of any features of OA.

#### Biceps tenosynovitis

Non-displaceable or non-fully compressible hypoechoic thickening of the long head of biceps tendon sheath with or without power Doppler signal of evidence of tendonopathy (see 9) or thinning of the long head of biceps tendon.

#### Tendinopathy

Hypoechogenicity or heterogeneity with thickening of the rotator cuff tendons compared to the other rotator cuff tendons of the ipsilateral or contralateral shoulder.

### Latent Class Analysis

### Methods

Each of the pathologies recorded during the scan, coded as present (2) or absent (1), was included in the model, together with covariates age and sex. A rho prior value of 1 was set to stabilise estimates. The Bayesian Information Criterion (BIC) value was chosen to determine the optimum number of classes present because it has been demonstrated to be the best performing of the available information criteria, preferred over Akaike’s Information Criterion (AIC), consistent AIC or adjusted BIC [1]. Initial models used two hundred random starts to investigate whether the optimum number of classes was stable and to identify the optimum seed for the final model. Missing data were assumed to be missing at random (MAR) and for patients with missing pathology scores the model estimates were adjusted to include only the observed items (full information maximum likelihood).

The maximum posterior probability of membership observed across the different classes was used to determine the most likely class membership for each patient. Having assigned patients to classes (hereafter groups) on this basis, for each group the corresponding mean posterior probability was calculated to give an indication of the accuracy of group assignment.

**Results**

The LCA suggested four or five groups existed. Both solutions were stable and had similar associated Bayesian information criterion (BIC) values. We interpreted the four group solution to be: bursitis with limited inflammation elsewhere (group 1); bursitis with extensive inflammation (group 2); RC tears (group 3); limited pathology (group 4). Groups were more clearly defined in patients with >90% probability of membership of their assigned group (Table S2). In the five group solution, groups 1, 3 and 4 remained largely unchanged, but group 2 split into patients with bursitis, subacromial impingement and tendinopathy (2a) and patients with tendinopathy in the absence of impingement, the majority of whom had bursitis (2b) (Table S3). At a meeting of 2 shoulder surgeons, 3 musculoskeletal radiologists and 2 rheumatologists, the four group solution was considered by the attendees to have face validity. The five group solution was felt to be less clinically meaningful and at risk of over-fitting the data.

**References**

1. Nylund KL, Asparouhov T, Muthén BO. Deciding on the number of classes in latent class analysis and growth mixture modelling: A Monte Carlo simulation study. Structural Equation Modelling. 2007;14(4):535-569.

### Table S1: Bayesian Information Criterion values for latent class analysis models with different numbers of latent classes

| Number of classes | BIC | Optimum seed | % of seeds associated with best model | Decision |
| --- | --- | --- | --- | --- |
| 2 | 1885.3 | 870700251 | 35% | Reject |
| 3 | 1027.6 | 2057177489 | 100% | Reject |
| 4 | 730.2 | 292175518 | 100% | Possible solution |
| 5 | 713.3 | 865172956 | 85% | Possible solution |
| 6 | 764.9 | 614998009 | 50% | Reject |

### Table S2: Four class solution (restricted to >90% probability of class membership)

|  | Bursitis (limited inflammation)  n=863 | Bursitis (extensive inflammation)  n=226 | RC tear  n=374 | Limited pathology  n=459 |
| --- | --- | --- | --- | --- |
| % of sample | 45 | 12 | 19 | 24 |
| Age, years: mean (SD) | 44.8 (11.0) | 67.0 (10.9) | 71.0 (10.7) | 44.9 (13.3) |
| Female: % | 53 | 51 | 52 | 48 |
| Pain duration, months*: median (IQR) | 6 (3, 10) | 5 (3, 9) | 5 (3, 7) | 4 (3, 8) |
| Steroid injection at time of scan: % | 44 | 45 | 11 | 23 |
| RC tear (y/n): % | <1 | 7 | 100 | 3 |
| (FT tear: % | - | <1 | 97 | - |
| Bursitis: % | 100 | 94 | 12 | <1 |
| Impingement: % | 100 | 55 | 98 | 3 |
| Calcific tendinitis: % | 12 | 22 | 3 | 9 |
| ACJ degeneration: % | 21 | 91 | 53 | 10 |
| Glenohumeral OA: % | <1 | 19 | 20 | 1 |
| Adhesive capsulitis: % | - | 3 | - | 13 |
| Biceps tenosynovitis: % | <1 | 48 | 8 | 1 |
| Rotator cuff tendinopathy: % | 13 | 96 | 16 | 17 |

*n=351; 72; 159; 181

### Table S3: Five class solution from latent class analysis

|  | Bursitis (limited inflammation)  n=1291 | Inflammation (impingement) n=448 | Inflammation  (no impingement)  n=306 | RC tear  n=523 | Limited pathology  n=432 |
| --- | --- | --- | --- | --- | --- |
| % of sample | 43 | 15 | 10 | 18 | 14 |
| Age, years: mean (SD) | 46.8 (11.2) | 64.0 (10.1) | 65.0 (10.6) | 69.0 (11.3) | 43.1 (11.9) |
| Female: % | 54 | 54 | 45 | 52 | 48 |
| Pain duration, months*: median (IQR) | 5 (3, 10) | 5 (3, 9) | 5 (3, 10) | 5 (3, 9) | 4 (3, 8) |
| Steroid injection at time of scan: % | 44 | 33 | 41 | 12 | 23 |
| RC tear (y/n): % | 3 | 26 | 20 | 99 | 6 |
| (FT tear: % | 1 | 20 | - | 90 | <1 |
| Bursitis: % | 98 | 100 | 57 | 28 | <1 |
| Impingement**: % | 86 | 98 | <1 | 95 | 3 |
| Calcific tendinitis: % | 14 | 16 | 18 | 5 | 8 |
| ACJ degeneration: % | 23 | 91 | 65 | 52 | 7 |
| Glenohumeral OA: % | <1 | 7 | 13 | 17 | <1 |
| Adhesive capsulitis: % | <1 | 2 | 9 | <1 | 12 |
| Biceps tenosynovitis: % | <1 | 30 | 9 | 9 | <1 |
| Rotator cuff tendinopathy: % | 23 | 85 | 77 | 21 | 16 |
| Probability of membership: mean | 0.88 | 0.80 | 0.75 | 0.90 | 0.91 |

*n=515; 106; 151; 219; 174 **n=1287; 446; 305; 522; 432

### Table S4: Comparison of 4 group solutions obtained in questionnaire respondents without RA (n=690)

|  | Re-calculated group excluding RA | | | |  |
| --- | --- | --- | --- | --- | --- |
| Original group | 1 | 2 | 3 | 4 | Total |
| 1 | 238 | 13 | 0 | 40 | 291 |
| 2 | 3 | 160 | 0 | 14 | 177 |
| 3 | 0 | 9 | 113 | 0 | 122 |
| 4 | 8 | 0 | 0 | 92 | 100 |
| Total | 249 | 182 | 113 | 146 | 690 |

Restricting the LCA to questionnaire respondents without RA (n=690 out of a total of 777 respondents), minimum adjusted BIC values indicated that a four (adjusted BIC=342) or five (adjusted BIC=324) group solution might exist. Using the four group solution, patients were generally assigned to the same groups they had been assigned to when the LCA was run in the full cohort (exact agreement over group assignment in 87% of cases); the five group solution was almost exactly the same as the four group solution for groups 1-3, but a limited number (n=17) of patients with adhesive capsulitis were moved from group 4 to form a separate group. The existence of this putative fifth group, which only represented 2% of the sample, should be confirmed in a prospective study with tighter inclusion criteria and standardised reporting of pathology. We opted to retain the four class solution to avoid overfitting the data at this stage.

### Table S5: Questionnaire results from all respondents, excluding those reporting rheumatoid arthritis (n=690)

|  | All respondents | N with data |
| --- | --- | --- |
| Age, years: mean (SD) | 56.0 (13.7) | 690 |
| Female: % | 54 | 690 |
| Smoker: % | 39 | 635 |
| Comorbidity count: median (IQR) | 1 (0, 2) | 553 |
| Painful sites count (including target): median (IQR) | 3 (1, 6) | 690 |
| Follow-up, months: median (IQR) | 25 (22, 29) | 684 |
| Had shoulder fracture before scan: (%) | 1 | 671 |
| Had shoulder dislocation before scan: (%) | 1 | 672 |
| Had breast/shoulder cancer before scan: (%) | <1 | 671 |
| Had major injury to target shoulder before scan: (%) | 9 | 670 |
| Had shoulder fracture since scan: (%) | <1 | 669 |
| Had shoulder dislocation since scan: (%) | <1 | 668 |
| Had breast/shoulder cancer since scan: (%) | <1 | 667 |
| Had major injury to target shoulder since scan: (%) | 3 | 670 |
| Had physiotherapy since scan: (%) | 62 | 630 |
| Had injection since scan: (%) | 67 | 639 |
| Had >1 injection since scan: (%) | 29 | 629 |
| Had surgery since scan: (%) | 22 | 620 |
| Still has shoulder pain: (%) | 66 | 673 |
| If still in pain: |  |  |
| Pain duration, months: median (IQR) | 24 (12, 36) | 366/442 |
| Has pain free periods: (%) | 73 | 435/442 |
| Experiences pain on moving in a certain way: (%) | 91 | 435/442 |
| If not still in pain: |  |  |
| How long since last had pain, months: median (IQR) | 12 (6, 18) | 175/231 |
| How long did pain last, months: median (IQR) | 10 (4, 18) | 198/231 |
| Symptoms at time of questionnaire (all respondents): |  |  |
| SPADI pain: median (IQR) | 36 (6, 64) | 678 |
| SPADI difficulty: median (IQR) | 18 (0, 46) | 674 |
| SPADI total: median (IQR) | 28 (5, 53) | 672 |
| Shoulder activity score: median (IQR) | 6 (3, 10) | 640 |
| EQ5D health index score: median (IQR) | 0.8 (0.6, 0.8) | 648 |
| EQ5D VAS: median (IQR) | 75 (60, 90) | 681 |
| EQ5D anxiety or depression (>0): (%) | 33 | 663 |
| Depression reported in comorbidity list: (%) | 16 | 628 |
| Difficulty standing from sitting (>1): (%) | 17 | 674 |

*Date of questionnaire completion missing **In patients who still had shoulder pain

### Table S6: Ultrasound findings for the four pathology groups restricted to patients who responded to the survey (n=690)

|  | Bursitis (limited inflammation)  n=291 | Bursitis (extensive inflammation)  n=177 | RC tear  n=122 | Limited pathology  n=100 |
| --- | --- | --- | --- | --- |
| % of sample | 42 | 26 | 18 | 14 |
| Pain duration, months*: median (IQR) | 6 (3, 10) | 5 (3, 10) | 5 (3, 8) | 4 (3, 8) |
| Steroid injection at time of scan: % | 46 | 42 | 14 | 31 |
| RC tear: % | 1 | 27 | 100 | 8 |
| Full thickness RC tear: % | <1 | 16 | 76 | - |
| Bursitis: % | 100 | 97 | 28 | 3 |
| Impingement: % | 86 | 68 | 92 | 8 |
| Calcific tendinitis: % | 14 | 16 | 5 | 18 |
| ACJ degeneration: % | 28 | 88 | 57 | 16 |
| Glenohumeral OA: % | <1 | 8 | 13 | - |
| Adhesive capsulitis: % | <1 | 3 | <1 | 17 |
| Biceps tenosynovitis: % | 2 | 22 | 13 | 1 |
| Rotator cuff tendinopathy: % | 23 | 95 | 20 | 17 |
| Probability of membership: mean | 0.86 | 0.81 | 0.89 | 0.94 |

*n=118; 60; 57; 35

### Table S7: Therapies received according to the presence of individual ultrasound-detected pathologies

|  | Steroid injection  (at scan) | Steroid injection  (since scan) | Physiotherapy | Surgery |
| --- | --- | --- | --- | --- |
| Number with data | 690 | 639 | 630 | 620 |
| RC tear Absent  Present | 45  14 | 72  50 | 62  63 | 19  32 |
| Bursitis Absent  Present | 22  43 | 53  72 | 59  63 | 22  22 |
| Impingement Absent  Present | 46  33 | 72  64 | 60  63 | 17  24 |
| Calcific tendinitis Absent  Present | 38  36 | 67  67 | 61  72 | 21  31 |
| ACJ degeneration Absent  Present | 38  37 | 67  67 | 65  59 | 23  22 |
| Glenohumeral OA Absent  Present | 38  19 | 67  57 | 62  60 | 23  11 |
| Adhesive capsulitis Absent  Present | 38  33 | 67  68 | 62  59 | 23  9 |
| Biceps tenosynovitis Absent  Present | 37  43 | 67  64 | 62  60 | 22  21 |
| Other tendinopathy Absent  Present | 36  39 | 66  69 | 60  65 | 22  22 |

All values in the table are %

### Table S8: Numbers of patients with data available for each survey item, by pathology group

|  | Bursitis (limited inflammation)  n=291 | Bursitis (extensive inflammation)  n=177 | RC tear  n=122 | Limited pathology  n=100 |
| --- | --- | --- | --- | --- |
| Smoker: | 270 | 161 | 108 | 96 |
| Comorbidity count: median (IQR) | 238 | 135 | 88 | 92 |
| Painful sites count (including target): median (IQR) | 291 | 177 | 122 | 100 |
| Follow-up, months: median (IQR) | 289 | 177 | 121 | 97 |
| Had shoulder fracture before scan: | 288 | 171 | 116 | 96 |
| Had shoulder dislocation before scan: | 288 | 170 | 118 | 96 |
| Had breast/shoulder cancer before scan: | 288 | 169 | 118 | 96 |
| Had major injury to target shoulder before scan: | 282 | 172 | 119 | 97 |
| Had shoulder fracture since scan: | 284 | 171 | 116 | 98 |
| Had shoulder dislocation since scan: | 286 | 168 | 116 | 98 |
| Had breast/shoulder cancer since scan: | 284 | 169 | 116 | 98 |
| Had major injury to target shoulder since scan: | 284 | 168 | 119 | 99 |
| Had physiotherapy since scan: | 260 | 159 | 115 | 96 |
| Had injection since scan: | 274 | 160 | 110 | 95 |
| Had >1 injection since scan: | 274 | 160 | 110 | 95 |
| Had surgery since scan: | 252 | 169 | 109 | 90 |
| Still has shoulder pain: | 287 | 171 | 117 | 98 |
| If still in pain: |  |  |  |  |
| Pain duration, months: median (IQR) | 153/181 | 87/108 | 70/90 | 56/63 |
| Has pain free periods: | 176/181 | 108/108 | 89/90 | 62/63 |
| Experiences pain on moving in a certain way: | 179/181 | 107/108 | 88/90 | 61/63 |
| If not still in pain: |  |  |  |  |
| How long since last had pain, months: median (IQR) | 84/106 | 47/63 | 20/27 | 24/35 |
| How long did pain last, months: median (IQR) | 92/106 | 54/63 | 21/27 | 31/35 |
| Symptoms at time of questionnaire: |  |  |  |  |
| SPADI pain: median (IQR) | 287 | 170 | 121 | 100 |
| SPADI difficulty: median (IQR) | 282 | 171 | 121 | 100 |
| SPADI total: median (IQR) | 282 | 169 | 121 | 100 |
| Shoulder activity score: median (IQR) | 271 | 162 | 111 | 96 |
| EQ5D health index score: median (IQR) | 271 | 168 | 114 | 95 |
| EQ5D VAS: median (IQR) | 287 | 174 | 121 | 99 |
| EQ5D anxiety or depression (>0): | 276 | 171 | 117 | 99 |
| Depression reported in comorbidity list: | 270 | 155 | 106 | 97 |
| Difficulty standing from sitting (>1): | 282 | 175 | 118 | 99 |

### Figure S1: Joints reported to be painful at the time of the survey, according to whether or not they were on the same side as the target shoulder

### Figure S2: Total SPADI scores according to whether patient had surgery between scan and survey
