# Supplementary figures and images for: Ultrasound-detected pathologies cluster into groups with different clinical outcomes: data from 3000 community referrals for shoulder pain
Source: Arthritis Res Ther. 2017 Feb 10;19:30. doi: 10.1186/s13075-017-1235-y (PMC5304553; doi:10.1186/s13075-017-1235-y)

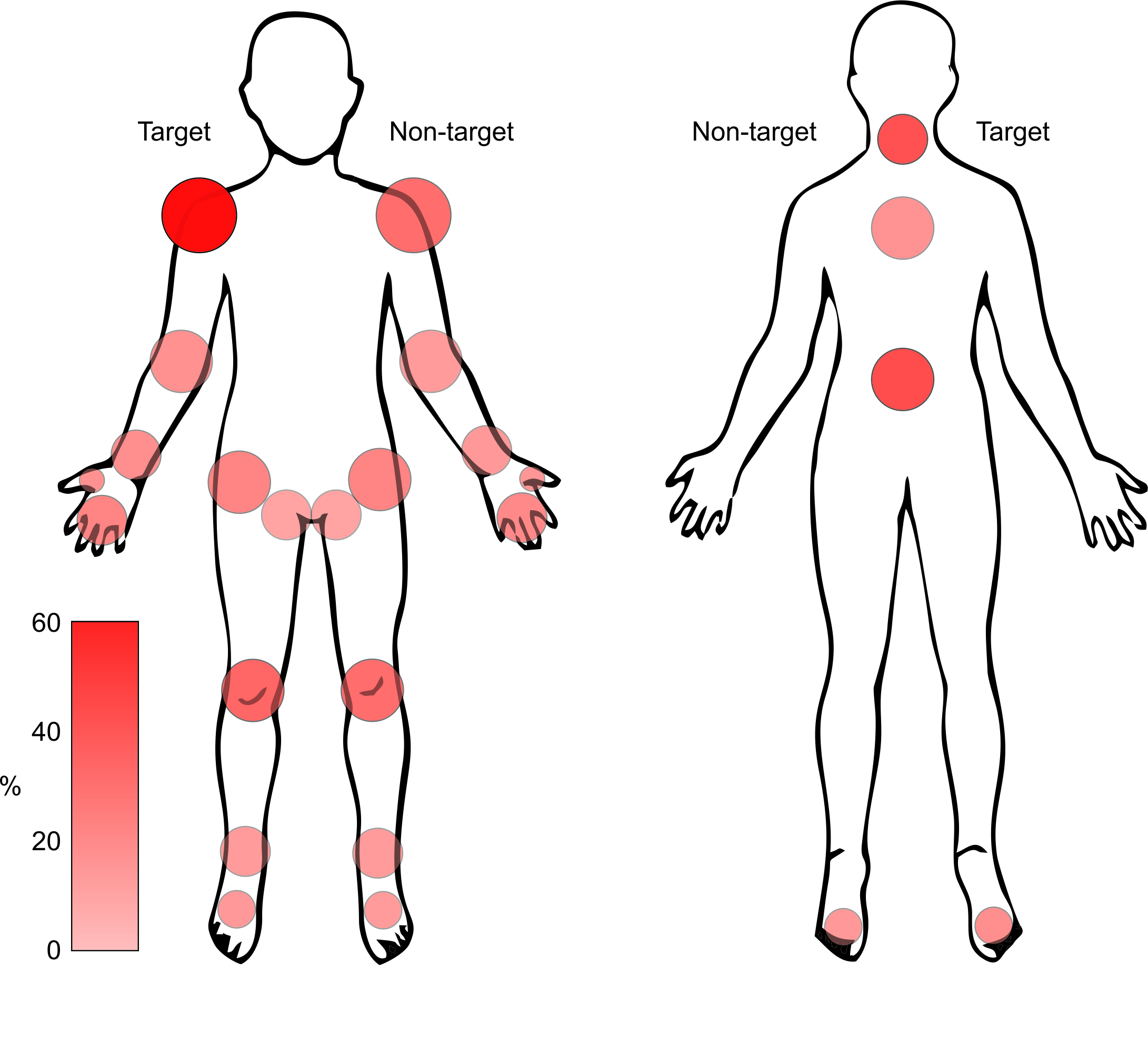

Supplement: Supplementary file 2 — Joint pain reported. (JPG 2553 kb) [file 13075_2017_1235_MOESM2_ESM.jpg]

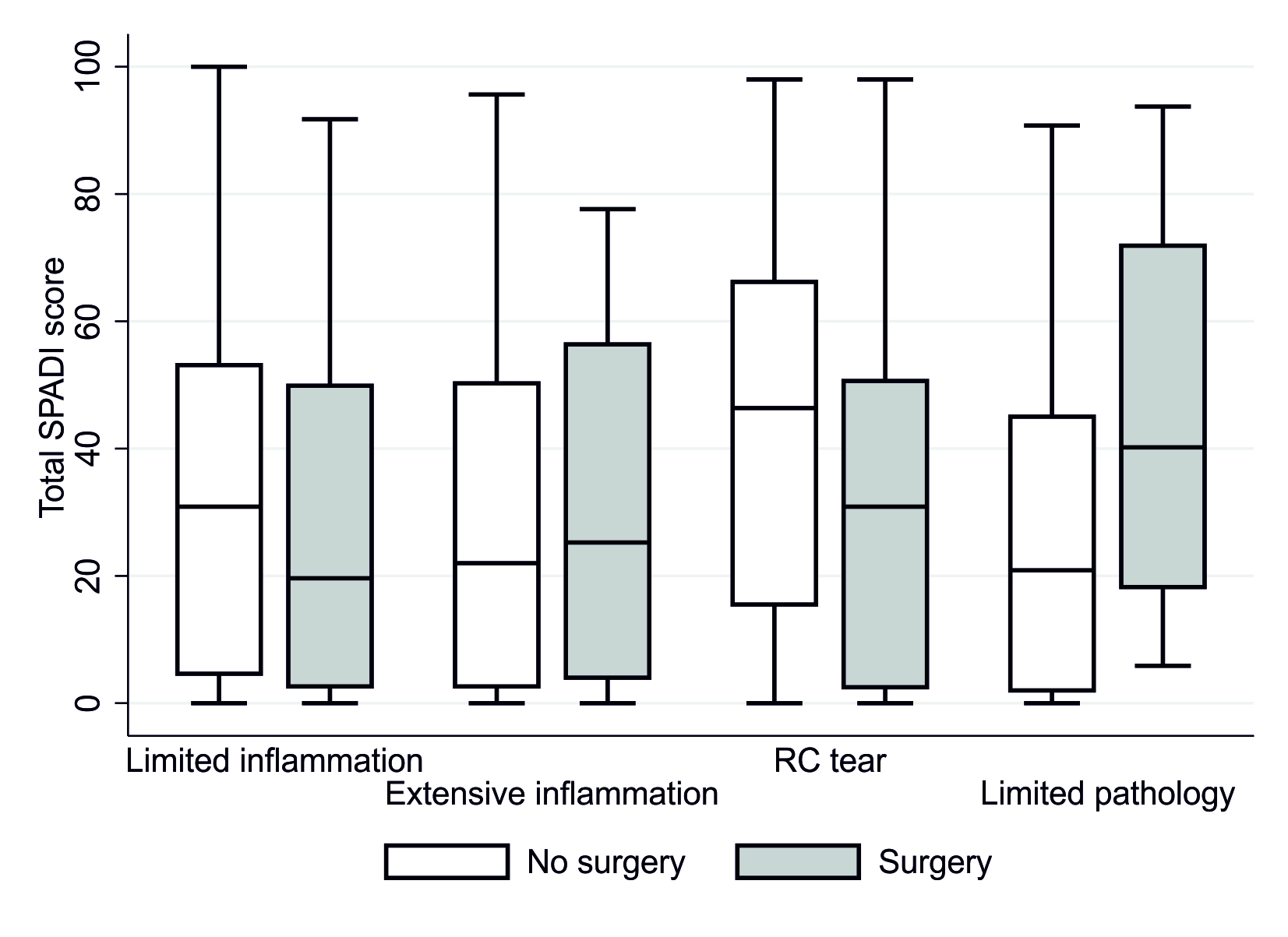

Supplement: Supplementary file 3 — Total SPADI by surgery. (JPG 2552 kb) [file 13075_2017_1235_MOESM3_ESM.jpg]
